# Dr. J. T. Bettis

**Published:** 1915-01

**Authors:** 


**Dr. J. T. Bettis,** Cleveland Homoeopathic College 1869, died at his home in Livonia, November 20, 1914. He was born in Albion, August 13, 1845. He served in Co. K, 54th Regt. N. Y. Volunteers, from July 26 to November 10, 1864. He studied with the late Dr. J. M. Dake of Albion before entering college. He located in Livonia Center in 1870 and in the village of Livonia in 1872. In 1876 he took a post graduate course in eye and ear diseases in the N. Y. Homoeopathic College. He was a founder of Livonia Masonic Lodge in which he had held several positions of honor. He was also a charter member of E. S. Gilbert Post, G. A. R., and had been commander of the Post for fourteen years at the time of his death. He established the first telephone line in Livonia. He was village health officer at the time of his death.

